# Metabolic State Alters Economic Decision Making under Risk in Humans

**DOI:** 10.1371/journal.pone.0011090

**Published:** 2010-06-16

**Authors:** Mkael Symmonds, Julian J. Emmanuel, Megan E. Drew, Rachel L. Batterham, Raymond J. Dolan

**Affiliations:** 1 Wellcome Trust Centre for Neuroimaging, Institute of Neurology, University College London, London, United Kingdom; 2 UCL Division of Medicine, Department of Metabolism and Experimental Therapeutics, University College London, London, United Kingdom; Yale University, United States of America

## Abstract

**Background:**

Animals' attitudes to risk are profoundly influenced by metabolic state (hunger and baseline energy stores). Specifically, animals often express a preference for risky (more variable) food sources when below a metabolic reference point (hungry), and safe (less variable) food sources when sated. Circulating hormones report the status of energy reserves and acute nutrient intake to widespread targets in the central nervous system that regulate feeding behaviour, including brain regions strongly implicated in risk and reward based decision-making in humans. Despite this, physiological influences per se have not been considered previously to influence economic decisions in humans. We hypothesised that baseline metabolic reserves and alterations in metabolic state would systematically modulate decision-making and financial risk-taking in humans.

**Methodology/Principal Findings:**

We used a controlled feeding manipulation and assayed decision-making preferences across different metabolic states following a meal. To elicit risk-preference, we presented a sequence of 200 paired lotteries, subjects' task being to select their preferred option from each pair. We also measured prandial suppression of circulating acyl-ghrelin (a centrally-acting orexigenic hormone signalling acute nutrient intake), and circulating leptin levels (providing an assay of energy reserves). We show both immediate and delayed effects on risky decision-making following a meal, and that these changes correlate with an individual's baseline leptin and changes in acyl-ghrelin levels respectively.

**Conclusions/Significance:**

We show that human risk preferences are exquisitely sensitive to current metabolic state, in a direction consistent with ecological models of feeding behaviour but not predicted by normative economic theory. These substantive effects of state changes on economic decisions perhaps reflect shared evolutionarily conserved neurobiological mechanisms. We suggest that this sensitivity in human risk-preference to current metabolic state has significant implications for both real-world economic transactions and for aberrant decision-making in eating disorders and obesity.

## Introduction

Prospect Theory, one of the most influential descriptive theories of decision-making under risk, emphasises that risk-attitude in humans is reference-dependent [1]. When choosing between options yielding gains, humans are on average risk-averse (i.e. avoiding options with a higher uncertainty or variance), while when choosing between options yielding losses below a reference point, humans make riskier choices. This finding is paralleled by observations in animals, where sensitivity to risk is systematically influenced by a metabolic reference point. For example, animals become more risk-seeking following a reduction in energy levels by fasting, or increase in basal energy requirements through change in ambient temperature [2], [3]. Stochasticity is ubiquitous in natural environments, and risk-sensitivity reflects a phylogenetically conserved adaptation, where maintenance of adequate nutrition and energy stores in the face of this environmental variability is critical for survival and reproduction [4], [5], [6], [7], [8].

Circulating hormones report the status of body energy reserves (e.g. adipose tissue), energy requirements, and acute nutrient intake to targets in the central nervous system that regulate feeding behaviour, including brain regions implicated in human decision-making [9], [10], [11], [12], [13]. There is therefore a potential for changes in metabolic state, and induced changes in hormone levels, to directly influence decisions in the economic domain.

Here, we sought to characterise whether changes in metabolic state systematically influence human risk-attitude in financial decisions. Do we observe consistent changes in risk-preference in the economic domain after feeding (i.e. a transfer of effect from the metabolic to the cognitive domain)? Hormones including oxytocin and testosterone levels have been shown to have an influence on economic behaviour [14], [15]. However, physiological state-dependent influences play no part in traditional economic theory, in contrast to ecological theory with its emphasis on a dependence of foraging behaviour on metabolic state [16].

Stephens suggested that when an animal chooses between two foraging options giving normally-distributed energetic returns with equal means but different variance, an organism aiming to maximise ‘fitness’ (i.e. survival probability) prefers safer (lower variance) options when above a metabolic reference point (e.g. energetic requirement over the day) but riskier (higher variance) options when below a metabolic reference point [17]. Alternative models predict that risk-preference will dynamically adjust depending upon metabolic state, energy reserves, and intake rate [18], [19]. If energy intake rate is below a reference point, this induces greater risk-seeking. Above a reference point, there is a change toward greater risk-aversion. The metabolic reference point is often taken in ecology as the intake rate required to reach a survival threshold, with the increasing probability of starvation as current intake rate drops below threshold promoting risk-seeking behaviour. This reference point can also be a reproductive threshold [20], or an alternative homeostatic marker set at the expected intake level according to previous feeding history [21], [22]. These models also predict that baseline risk-attitude will depend upon baseline energy reserves, with increased baseline risk-aversion as energy reserves exceed a threshold. Finally, at repletion, marginal changes in energy are not predicted to have significant impact on ecological fitness, and organisms will become insensitive to risk (risk-neutral). This relationship between energy intake, energy reserves, and attitude toward risk closely mirrors Prospect Theory's account of the relationship between risk-attitude for money, economic reference points, and the effect of changes in wealth (see Figure S1 for illustration). Indeed, a direct link between these conceptual frameworks from psychology and ecology is suggested by observations that human monetary decisions under risk are systematically influenced by an ‘earnings budget’, and that risk-preference in starlings changes according to relative amounts of gain or loss in food reward, even when overall nutritional intake is controlled (i.e. experimental manipulations of the reference point) [22], [23].

We tested risky decision-making in healthy men over three sessions, one week apart, using a within-subjects randomised design. We employed a controlled feeding manipulation, and assayed the same individual's decision-making preferences across different metabolic states; post-14 hr fast, immediately following and one hour post-ingestion of a 2066 kcal meal. We assessed the effect of our feeding paradigm on subjective measures of appetite as well as circulating levels of acyl-ghrelin, with percentage body fat and circulating leptin levels providing an assay of energy reserves. Leptin is a peptide hormone modulating satiety and indexing adiposity [24]. Prandial suppression of circulating acyl-ghrelin, the primary centrally-acting orexigenic hormone, is a humoral signal of acute nutrient intake highly sensitive to short-term changes in metabolic state, correlating with subjective indices of hunger [25].

We predicted that individuals making monetary decisions would become more risk-averse after feeding if the meal had a larger impact on metabolic state (i.e. a larger fall in ghrelin). This effect should only occur at the time when ghrelin levels fall, as there is a time-lag before the calorific impact of a meal is registered in terms of changes in plasma hormone concentrations. We hypothesised that there might also be an immediate shift towards risk-neutrality due to satiation (a non-humoural, rapid effect), as ecological models predict a shift towards a risk-neutral attitude with repletion.

## Materials and Methods

### Subjects

Twenty four, healthy, normal-weight, male volunteers were recruited (mean age: 25±7 years; BMI: 22.6±1.7 kg/m2; Table S1). One subject was excluded because of baseline fasting hyperglycaemia, another dropped out after the first week, and three excluded because of technical problems. Thus, 19 subjects' data were included in the final behavioural analysis. From these, one subject had haemolysed blood samples for a relevant timepoint, which renders hormonal assay inaccurate, and is excluded from the endocrine analyses. Volunteers provided informed consent and this study was approved by the University College London Research Ethics Committee.

### Study Protocol

**Figure 1:**
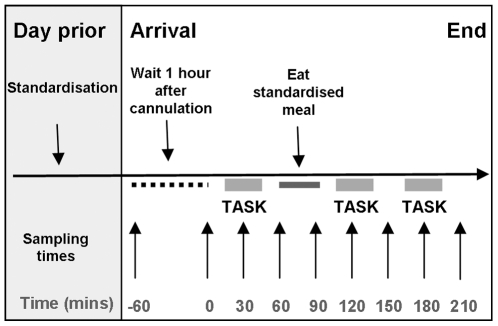
Sequence of each experimental session. Testing was performed at fasting (t = 0 to 60 mins), just after a meal (t = 90 to t = 150 mins), and 1 hr after feeding (t = 150 to t = 210 mins) Hormonal assays and visual analogue scale ratings were taken every 30 mins.

Participants attended a preliminary session, where anthropometric measurements were taken (height with a stadiometer, weight and percentage body fat with Tanita scales (Tanita, Hoofdrrop, Netherlands), and subjects received verbal and written information familiarizing them with the experimental procedure and visual analogue scores (VAS). VAS assessed hunger, fullness, prospective food consumption, sickness and anxiety [26], [27], and were 100 mm long with positive and negative text ratings anchored at each end. The day before testing sessions, subjects followed a standardization protocol [28], involving refraining from alcohol and strenuous exercise and consuming an 774 kcal meal between 19:30 and 20:30. Subjects then fasted and drank only water until attending our clinical facility the following morning. On each study day subjects arrived at 9:00 and an ante-cubital arm vein was cannulated (t = −60 min) for subsequent blood sampling. After relaxing for one hour post-cannulation, baseline blood samples were taken and subjects completed visual analogue scores (VAS) (t = 0 min). Blood samples were drawn and subjects completed VAS, every 30 minutes from t = 0 until t = 210 min. At t = 60 min subjects consumed a standardized 2066 kcal meal within 30 mins (Figure 1 and File S1).

Testing was undertaken in three different feeding states: fasted (t = 0 to t = 60 min), immediately post-meal (t = 90 to t = 150 min) and 60 minutes post-meal (t = 150 to t = 210 min). Subjects performed one of three different decision-making tasks within each hour to ensure that cognitive demand was the same throughout the experimental session. Each task was performed once in each week, in randomised order. These comprised a risk-preference elicitation task using paired lotteries (see below), and two additional tasks (see File S1). Each task took approximately 30 +/−5 mins to complete. Importantly, behavioural measures were correlated with hormone levels and VAS from the nearest 30 min sampling point, ensuring that assay titres corresponded with an accurate reflection of hormonal status whilst performing the cognitive task.

### Risk-preference Paradigm

**Figure 2:**
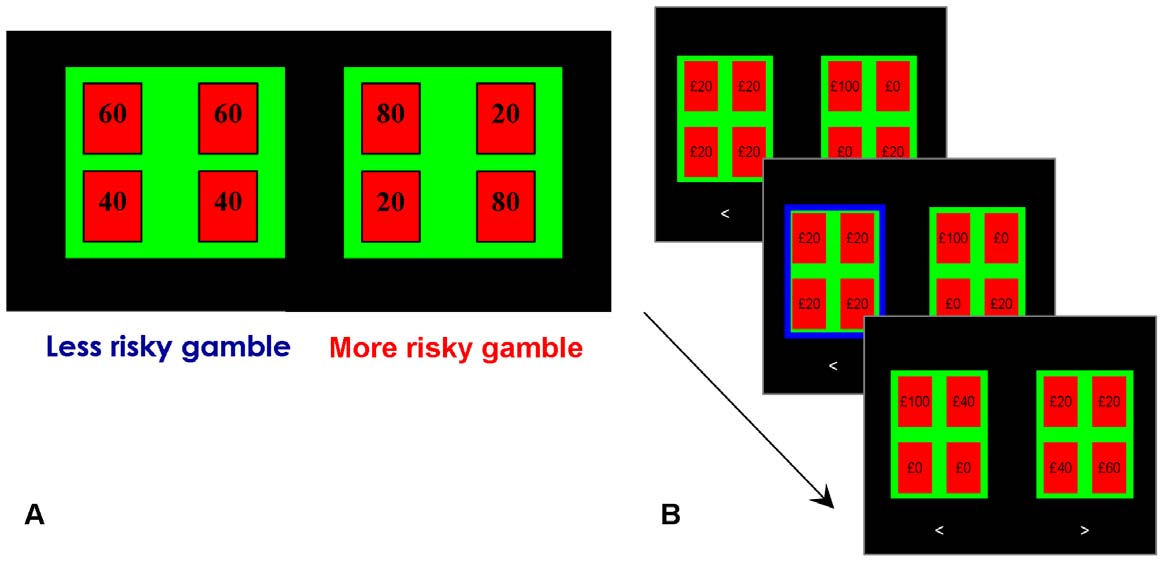
Risk preference task. A. On every trial, a choice between two lotteries was presented on-screen, and subjects were required to select their preferred option from each pair. Lotteries were represented as four cards, with a numerical display of one of six fixed monetary prizes (£0, £20, £40, £60, £80, £100). Each card had an equal chance of being picked. B. The same set of 200 sequential paired lotteries were presented on each visit. Subjects had unlimited time to make a button-press response – the selected lottery was then highlighted on screen with a blue border, before the next trial ensued. No feedback was given about lottery outcomes during the task.

We employed a multiple paired lottery choice task, presenting a sequence of 200 paired lotteries (Figure 2; Table S2), with subjects required to select one preferred option per pair [29]. Lotteries were constructed by varying the probabilities over six fixed monetary prizes (£0, £20, £40, £60, £80, £100), represented as four cards with one of these amounts displayed upon each card. Thus, the probability of each prize could be varied in 0.25 increments (0, 0.25, 0.5, 0.75, 1). Each week, subjects were exposed to the same set of lotteries. The left-right on-screen position of the lotteries, and position of the 4 cards within each lottery were randomised, to ensure attention to the task, and to avoid response habituation. On debriefing, no subject reported realising that the lottery sequences were the same across the three weeks. The lottery list was constructed on the assumption that individuals are on average risk-averse – hence most offers were between a safer lottery with lower expected value (EV), and a riskier (higher variance) lottery with higher EV, allowing us to maximise power for discriminating small but consistent state-dependent differences in risk-preference within-subjects while maintaining the same lottery set across subjects. Lotteries were presented on a laptop computer screen, and keypress responses recorded using Cogent 2000 software (Wellcome Trust Centre for Neuroimaging, London).

### Behavioural analysis

Our primary measure was the percentage of riskier vs less risky choices made in each week by every subject. Risk was quantified by the variance of lottery prizes about the mean value [30], [31]. This percentage measure provides an indication of any consistent changes between metabolic states across subjects. As the sets of paired lotteries are identical across subjects and sessions, any differences between states reflect changes in decision criteria. We also implemented a logistic regression model to separately analyse changes in sensitivity to EV and variance across states. This enabled us to describe changes in risk-return tradeoff, estimate absolute risk-preferences, and the degree of choice noisiness. Statistical analysis was implemented in MATLAB (version 6.5, MathWork, Natick, MA), and SPSS (SPSS for Windows, Rel. 12.0.1. 2001. Chicago: SPSS Inc.). For one subject, an extended list of 360 paired lotteries was used for the first two sessions, and the reduced list of 200 lotteries used on session three. We excluded this subject when analysing choice percentages (as these will depend upon the set of choices), but included these data in model based analyses (as model parameter estimation is possible for either choice set).

### Decision-making model

In addition to the summary percentage of risky choice, we derived an absolute measure for risk-preference in each state by fitting a mean-variance logistic regression model (see Appendix S1).

## Results

### Metabolic state measures

**Figure 3:**
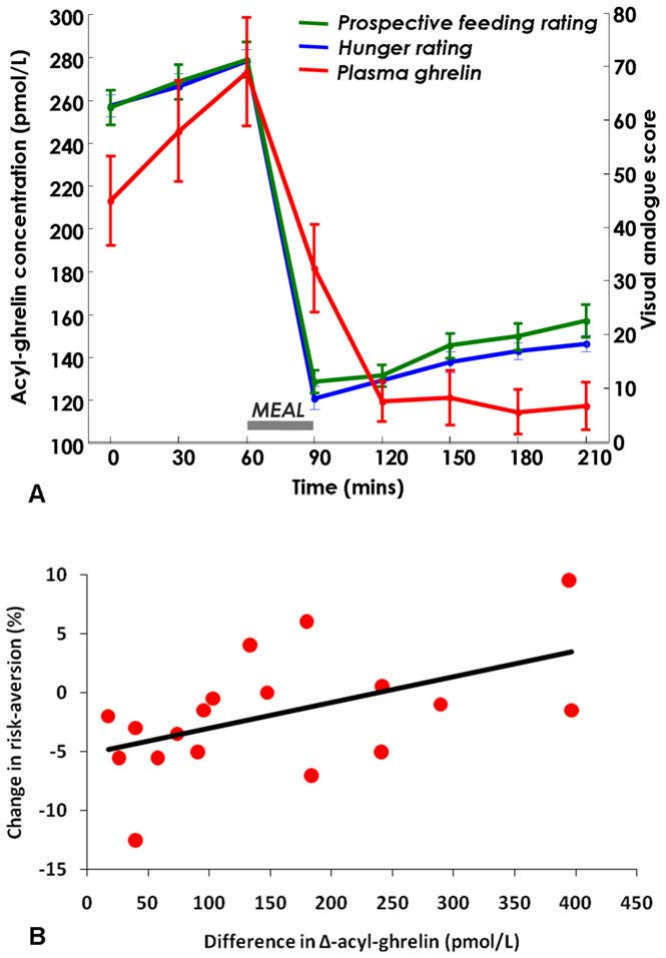
Metabolic state and change in risky choice. A. Change in hunger VAS (blue), prospective feeding VAS (green), and plasma acyl-ghrelin levels (red) over time-course of session, assayed every 30 mins, averaged across sessions and subjects. There was a significant drop in both hunger ratings and plasma acyl-ghrelin levels (p<0.001) after eating; the timecourse of this fall was slower for acyl-ghrelin, showing that this peripheral signal of acute nutrient intake is delayed. Error bars show s.e.m. B. Change in percentage of risky choices significantly correlated with the difference in Δ-ghrelin measurements (within week change in ghrelin from baseline), measured one hour after the meal. p = 0.022, r^2^ = 0.22 (N = 18).

Our paradigm was effective at manipulating subjective ratings of hunger and inducing significant concurrent changes in acyl-ghrelin levels (Figure 3A). There was a highly significant change in self-reported visual analogue scores (VAS) for hunger over the eight measured timepoints, before and after the meal, and across subjects (two-way repeated measures ANOVA (week, timepoint), main effect of timepoint: F(7,126) = 266, p<0.001). This effect was consistent across weeks (main effect of week: F(2,36) = 0.75, p = 0.48), although there was highly significant heterogeneity in the effect of the meal between subjects (F(1,18) = 571, p<0.001). Hunger VAS increased from baseline to administration of the meal (increase in hunger VAS from t = 0 to t = 60 min: 8.2±1.4, post-hoc contrast, t = 0 vs t = 60, F(1,18) = 36.9, p<0.001), then fell immediately post-meal, reaching a nadir at t = 120 min (decrease in hunger VAS from t = 0 to t = 120 min: 52.0±2.4, post-hoc contrast, t = 0 vs t = 120, F(1,18) = 462, p<0.001).

Meal consumption caused a significant drop in acyl-ghrelin levels (two-way repeated-measures ANOVA, main effect of timepoint: F(7,119) = 28.5, p<0.001), commensurate with the change in hunger ratings (average correlation between mean ghrelin and mean hunger VAS: Pearson's R = 0.79), which also peaked just before the meal (increase in plasma acyl-ghrelin from t = 0 to t = 60 min: 63.1±16.9 pmol/L, post-hoc contrast, t = 0 vs t = 60, F(1,17) = 14.0, p = 0.02), falling to trough level at t = 120 min (decrease in plasma acyl-ghrelin from t = 0 to t = 120 min: 98.7±16.5 pmol/L, post-hoc contrast, t = 0 vs t = 120, F(1,17) = 35.9, p<0.001). There was highly significant variation in the effect of the meal on acyl-ghrelin level changes (between-subjects effect: F(1,17) = 110.5, p<0.001). However, there was no significant within-subjects difference in hormonal profiles across weeks (main effect of week: F(34,2) = 0.50, p = 0.61), nor an interaction between week and timepoint (F(14,238) = 1.17, p = 0.30)

### Effect on risk-sensitive choice

**Figure 4:**
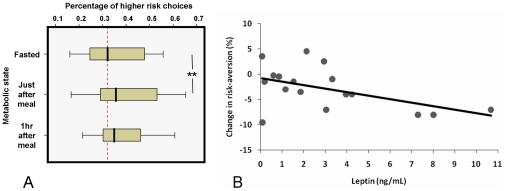
Change in risky choice and baseline adiposity. A. Y-axis shows percentage of trials (n = 200) where the riskier (higher variance) lottery was chosen in preference. Red dotted line indicates average baseline fasting percentage across subjects. Box indicates middle quartiles, bar widths show range. There was a significant decrease in risk-aversion (i.e. increase in risky choices) immediately after feeding (p = 0.007, N = 19). B. Leptin level (x-axis) against change in risk preference for each subject (just after meal – fasted baseline). Best-fitting least-squares estimated linear regression line shown in black (p = 0.046, r^2^ = 0.24).

Metabolic state significantly affected choice (one-way repeated-measures ANOVA, F(2, 34) = 3.22; p = 0.05 (sphericity assumed, Mauchly's W = 0.86, p = 0.30)), with a significant fall in risk-aversion immediately after eating (baseline fasted percentage risky choice  = 37.4%, s.e.m. = 3.0; within-subject increase in risky choice just after meal  = 2.8%, s.e.m. = 0.9%; F(1,17) = 9.50, p = 0.007; Figure 4A). This overall difference was no longer significant one hour post-feeding (F(1,17) = 2.48, p = 0.134). The difference between fasting and immediate post-meal was highly significant, irrespective of whether the lotteries were classified by variance (as above), standard deviation (paired t(17) = 3.08, p = 0.007), coefficient of variation (paired t(17) = 3.04, p = 0.007), or variance-to-mean ratio (paired t(17) = 3.00, p = 0.008).

The immediate impact of nutrient intake on risky choice showed a dependence upon baseline indices of body mass index (BMI), percentage body fat and circulating leptin concentrations. Higher baseline leptin correlated with an increase in riskier choices (i.e. a greater fall in risk-aversion) immediately after eating compared to the fasted state (F(1,17) = 4.75, p = 0.046, r^2^ = 0.24; Figure 4B). There was also a significant linear relationship between this change in risk attitude and both BMI (F(1,17) = 4.74, p = 0.046, r^2^ = 0.24), and percentage body fat (F(1,17) = 3.71, p = 0.073, r^2^ = 0.20).

The effect of the meal one hour post-feeding (measured by Δ-ghrelin, the within-week change in acyl-ghrelin from t = 0 min), significantly correlated with difference in risk attitude compared to baseline (F(1,17) = 6.56, p = 0.022, r^2^ = 0.22; Figure 3B). Greater prandial suppression of acyl-ghrelin concentrations, reflecting a larger impact of the meal on reducing a signal of hunger, led to a shift towards less risky choices. By contrast, a small effect correlated with a shift towards more risky choices. Crucially, this effect was only evident an hour after feeding once ghrelin levels had fallen (i.e. once the calorific impact of the meal had registered) (fasting vs just after eating: F(1,17) = 0.17; p = 0.69).

### Decision-making model

To quantify changes in risk-sensitivity, and demonstrate a selective effect of metabolic state on risk attitude, we fit individual subject behaviour to an economic decision-making model (see Appendix). The difference in risk between the two lotteries significantly influenced choice over and above the difference in EV alone, as a full model with both mean and variance terms was greatly superior to a reduced model where choices are based solely upon EV (likelihood ratio test, mean χ^2^(1) = 58, p<0.001). Subjects were all risk-averse at baseline (mean risk coefficient = 1.26×10^−2^; std = 0.96×10^−2^). The risk coefficient decreased significantly (indicating reduced risk-aversion) between fasting and one hour post-feeding (paired t(18) = 2.15; p = 0.039, one-tailed). We saw no difference in choice randomness in any state (fasted vs just fed: paired t(18) = 0.94, p = 0.35; fasted vs 1 hr: paired t(18) = 0.18, p = 0.86), indicating that feeding does not make choices more ‘noisy’. Change in risk coefficient across states significantly correlated with hormonal indices of baseline metabolic state and the meal-effect on hunger.

Confirming our initial analysis, leptin correlated with reduced risk-aversion immediately after eating compared to the fasted state (F(1,18) = 5.90, p = 0.027, r^2^ = 0.27). We also observed a significant correlation between this change in risk attitude and percentage body fat (F(1,18) = 4.59, p = 0.048, r^2^ = 0.22), and a trend in relation to BMI (F(1,18) = 4.23, p = 0.057, r^2^ = 0.21). Prandial suppression of acyl-ghrelin (t = 0 min to one hour post-feeding) correlated with a difference in the risk coefficient compared to baseline (F(1,18) = 6.62, p = 0.020, r^2^ = 0.29). Translating this effect size into financial terms indicates that, when fasted, subjects are predicted to be indifferent between a 50∶50 gamble of winning £30 or £0, and a sure amount of £8.45, giving a risk premium of £15-£8.64 = £6.55. Immediately after eating, for the same gamble, subjects are now indifferent to a sure amount of £9.40, a risk-premium of £5.50. Quantitatively, this indicates a decrease of £0.95 in risk premium for this lottery after feeding.

## Discussion

Changes in metabolic state systematically altered economic decision making. Individuals became more risk-averse with a greater post-prandial fall in acyl-ghrelin (i.e. larger signal of nutrient intake). A smaller effect, indicating a lower than anticipated impact of the meal, correlated with greater risk-seeking. This effect was only present an hour after eating, once ghrelin levels changed. This observation of an homeostatic dependence of choice upon metabolic state is consistent with ecological perspectives on risk [19], however a transfer of effect from the metabolic to the monetary domain has not been demonstrated previously. Importantly, these effects are therefore independent of baseline (economic) risk-attitude.

A direct comparison can be made with Prospect Theory, where changes in wealth below a reference point induce risk-seeking behaviour, while earnings above a reference point promote risk-aversion [32]. Similar reference-dependent change in risk attitude for food rewards has also been seen in animals [22]. Critically, this suggests that changes in acyl-ghrelin signal the effects of a caloric load (i.e. calorie intake rate) that are relative, or adapted to, metabolic requirements. In other words, the degree to which acyl-ghrelin changes after a meal could act as a hormonal signal for the adequacy of the current rate of calorific intake, and act centrally to modify behaviour. Mechanistically, ghrelin-receptors are expressed in neurons in hypothalamus, ventral tegmental area, and substantia nigra, which project to dopaminoceptive regions implicated in economic decision making under risk in humans [33]. These include amygdala and orbitofrontal cortex, regions implicated in reference-dependent valuation of losses and gains and the framing effect [34], [35].

We also see an immediate effect of a calorific load, with a fall in risk-aversion dependent upon baseline leptin levels. This can also be explained in this framework. The immediate impact of the meal (mediated through non-hormonal mechanisms) induces satiation, where further calorific intake is assessed to carry mimimal additional value. This induces risk-neutral behaviour. The predicted degree and direction of *change* in risk aversion depends upon baseline energy reserves. At reserve levels close to the reference point there is baseline risk-neutral behaviour (for food). As energy reserves rise above the reference point, there is baseline risk-aversion, because the relationship between fitness and energy is more concave (see Figure S1 for illustration). As observed, there is hardly any change in risk-aversion at lower energy reserves (adiposity), but a fall in risk-aversion if energy reserves are higher. Consistent with previous findings, adiposity is not correlated with baseline risk-attitude for money [36]. Instead, we find that it predicts change in risk-attitude from the fasted state to immediately after eating.

This effect occurs before the impact of the calorie load is perceived. Once the energetic impact of the meal registers as a change in ghrelin levels, the shift in risk-attitude is linked to endocrine feedback. The magnitude of these effects for an individual will depend upon a number of factors, in particular the precise shape of the relationship between the utility of food and baseline energy reserves in different metabolic states, which is also likely to be subject to considerable inter-individual variation. Additionally, the psychological mechanisms mediating changes in risk preference are not well understood, and some of the systematic behavioural changes we observe may reflect, for example, an influence of hunger and receipt of food on affect [37], [38]. Critically, we demonstrate a quantifiable and systematic link between physiologically measured metabolic state, and economic behaviour.

Prandial ghrelin suppression is reduced in obesity [39]. Thus, we predict greater risk-seeking in obese individuals following feeding, augmented by larger immediate post-prandial effects on risk-taking due to higher baseline adiposity. This mechanism may underpin a component of the aberrant decision-making seen in obese individuals, including impulsivity and reward-seeking behaviour [40], [41]. We also predict profound effects on decision-making for individuals operating at very low baseline energy reserves, and note such an explanation has been invoked to explain increased impulsivity in anorexia nervosa [42]. Finally, it is of interest that manipulations affecting hormonal responses to feeding, such as dieting (where circulating acyl-ghrelin increases), or bariatric surgery, may well have cognitive effects, including effects on decision making, beyond the metabolic domain [43].

Our demonstration that metabolic state influences human risky economic decisions is predicted by biological models accounting for metabolic reference points, but not by normative economic theory. It is tempting to speculate that maladaptive decision making in aberrant metabolic states may arise out hard-wired imperatives driving strategic decision-making adapted to deal with feeding decisions within a normal biological range. In the context of our study, biology would seem to inform economic theory, not only in providing explanations of psychological phenomena such as loss aversion, but also in highlighting substantive effects of state changes on economic decisions, perhaps reflecting shared evolutionarily conserved neurobiological mechanisms.

## Supporting Information

File S1 Supplementary Methods and Analysis.
(0.04 MB DOC)

Figure S1 Schematic of risk-attitude changes in relation to a reference point (either for money or for food/energy). Risk-attitude equates to the curvature of this relationship. Below reference point, risk-seeking behaviour is seen. Near the reference point, decisions are risk-neutral (insensitive to risk). As energy or wealth increases, increasing risk-aversion is seen. At very high levels (e.g. repletion or satiation), this relation saturates and we again see risk-neutral behaviour. Note: Above the reference point, the relationship between wealth/energy and utility/fitness is marginally decreasing (concave), engendering risk-aversion. This is because, for a concave function, the average ‘utility’ (i.e. average y-axis value) of any two outcomes always equates to less ‘wealth’ than the average wealth (i.e. average x-axis value) of the same two outcomes, by Jensen's inequality. This means that a sure amount with equivalent value to the average (mean) wealth of two outcomes will always be preferred to a gamble with 50∶50 chance of getting one or other outcome, thus such an individual is described as being averse to risk. A similar argument applies for risk-seeking being engendered by a convex relation between wealth/energy and utility/fitness.
(0.12 MB TIF)

Table S1 Baseline anthropometric and glucose results for included subjects.
(0.04 MB DOC)

Table S2 List of 200 lotteries used for risk preference elicitation (all amounts in pounds; risk difference in pounds^2^).
(0.45 MB DOC)

Appendix S1 Decision making model.
(0.02 MB DOC)
