# Supplementary material for: Metabolic State Alters Economic Decision Making under Risk in Humans
Source: PLoS One. 2010 Jun 16;5(6):e11090. doi: 10.1371/journal.pone.0011090 (PMC2886827; doi:10.1371/journal.pone.0011090)
Supplement: File S1 — Supplementary Methods and Analysis. (0.04 MB DOC) [file pone.0011090.s001.doc]

**SUPPLEMENTARY TEXT**

*Subjects*

Subjects were weight-stable for three months prior to recruitment. Exclusion criteria were the use of medications, any food allergies and presence of any medical or psychiatric illnesses. Volunteers provided informed consent and this study was approved by the University College London Research Ethics Committees.

Baseline measures of reward and food-related behaviour were normal (BIS: 17.3 ± 2.8; BAS drive: 10.0 ± 2.3; BAS reward responsiveness: 16.5 ± 1.4; BAS fun seeking: 11.4 ± 2.3; n = 19).

*Blood processing and assays*

Blood was collected into EDTA tubes containing 5000 kallikrein inhibitor units-per-ml of aprotonin (Bayer, Newbury, Berks, UK). Plasma was separated immediately by centrifugation at 4 °C. Samples for analysis of acyl-ghrelin were acidified by addition of 50µl of 1N hydrochloric acid (HCl) per ml and 4-(2-Aminoethyl)-benzenesulfonyl fluoride hydrochloride (Fluka, UK), 10µl 100mg/ml, was added. Samples were frozen and stored at -80^o^C until assayed.

For all assays samples were measured in duplicate. Fasting plasma leptin concentrations were measured using a commercially available ELISA (Millipore UK Ltd., Watford, UK). All samples were run on one plate and the sensitivity was 0.125 ng/ml and the intra-assay variation 3.5%. Plasma acyl-ghrelin was measured using commercially available radioimmunoassay (Millipore UK Ltd., Watford, UK). The assay sensitivity was 7.8 pg/ml, the intra- assay variability was 6.6% and the, inter-assay variability was 8.3%.

*Control Paradigms*

We used two additional cognitive paradigms in each week, controlling for cognitive load within each session. These comprised an intertemporal choice and a learning task. In the intertemporal choice task, subjects were required to make sequential choices between different amounts of money with different waiting times to payment. Subjects were presented with 200 choice pairs on a computer screen; 180 were between a smaller amount of money sooner and a larger amount of money after a delay, with the remaining 20 trials being the converse. Amounts ranged from £3-£100, and delays from 3 weeks-1 year. In the learning task, subjects had to learn about the likelihood of being rewarded from a set of 8 fractal stimuli, each giving 50p or 0p reward per trial with probability of either 0.8 or 0.2. This was repeated with a new set of fractal stimuli in week 2, and in week 3 subjects were shown paired stimuli from the first two sessions and asked to pick their preferential option, with additional monetary reward generated according the probabilistic contingencies of their chosen stimuli. All tasks were of approximately the same duration (30+/-5 mins), and in intervening periods subjects were able to rest or read while remaining in the study room.

*Payment*

Payout was determined by a random lottery incentive mechanism, with one choice, selected randomly across all 3 weeks, played out for real to determine winnings. A similar mechanism was used for the intertemporal choice task, with a provision that one of either the risk or the intertemporal choice task would be played out (chosen by random number generation on a computer). Winnings from the risk preference task ranged from £0-80, in addition to a baseline payment of £40/week for participation (generated from the rewards accrued in the cue learning task).

*Other visual analogue measures*

There was a highly significant concomitant change in prospective food consumption ratings over the course of each session (two-way repeated measures ANOVA (week, timepoint), main effect of timepoint: F(7,126) = 106, p<0.001; increase in prospective feeding VAS from t = 0 to t = 60 min: 9 ± 1.5, post-hoc contrast, t = 0 vs. t = 60, F(1,18) = 34.0, p <0.01; decrease in prospective feeding VAS from t = 0 to t = 120 min: 49.4 ± 4.2, post-hoc contrast, t = 0 vs t = 120, F(1,18) = 137, p < 0.001; average correlation between acyl-ghrelin and prospective food consumption VAS: Pearson’s R = 0.76).

*Acyl-ghrelin changes*

We calculated within-week changes in acyl-ghrelin from the t=0 min timepoint (Δ-ghrelin), which controls for small variations in fasting acyl-ghrelin levels between weeks, for each 30-minute interval throughout a session. Differences in Δ-ghrelin between weeks, calculated from the end of the 30 min interval in which each subject performed the task (t=30/60 min; t=120/150 min; t=180/210 min), indicate the relative difference in orexigenic drive between the three timepoints at which subjects performed the risk task.

Acyl-ghrelin levels significantly differed from the fasted state across subjects in the specific 30 min window when risk-preference was assessed, (one-way repeated measures ANOVA, F(2, 34) = 17.7, p<0.001). Pairwise comparisons reveal that this difference only became significant one hour post-prandially (within-subjects contrasts: immediately post-eating vs fasted, F(1, 17) = 0.228, p = 0.64; one-hour post-eating vs fasted, F(1,17) = 16.09, p = 0.001).

*Leptin analyses*

As leptin is produced by adipose tissue, we can expect a correlation between body fat and leptin levels, while BMI can be affected additionally by lean (muscle) mass. There is significant positive correlation between circulating plasma leptin concentrations and the body fat percentage measure (Pearson R = 0.55, p = 0.01, one-tailed), and a trend toward a correlation of BMI and leptin levels in our participants (Pearson R = 0.37, p = 0.06, one-tailed). There is also highly significant correlation between BMI and body fat percentage (Pearson R = 0.83, p <0.001). Given this, the addition of BMI to leptin as a predictive variable in a multiple linear regression explained an extra 9% variance in the change in risk attitude, although this was not significant (r2 change = 0.09, p = 0.20), while the addition of body fat percentage did not contribute further due to colinearity (r2 change < 0.01, p = 0.78). There was no correlation between baseline acyl-ghrelin and leptin levels (p = 0.15). Additionally, there was no correlation between leptin, body mass index, or body fat percentage and mean risk attitude across session (p>0.6 for all correlations).

The absence of a significant interaction between the baseline effect of leptin and the effect of an acyl-ghrelin change on risk aversion (2-way categorical ANOVA, leptin x acyl-ghrelin: F(1,13) = 0.88, p = 0.36), may indicate separate effects at different timescales (immediate vs delayed effect of feeding), although our sample is not powered to detect this interaction given that all our subjects were of normal weight.
