# Supplementary figures and images for: Metabolic State Alters Economic Decision Making under Risk in Humans
Source: PLoS One. 2010 Jun 16;5(6):e11090. doi: 10.1371/journal.pone.0011090 (PMC2886827; doi:10.1371/journal.pone.0011090)

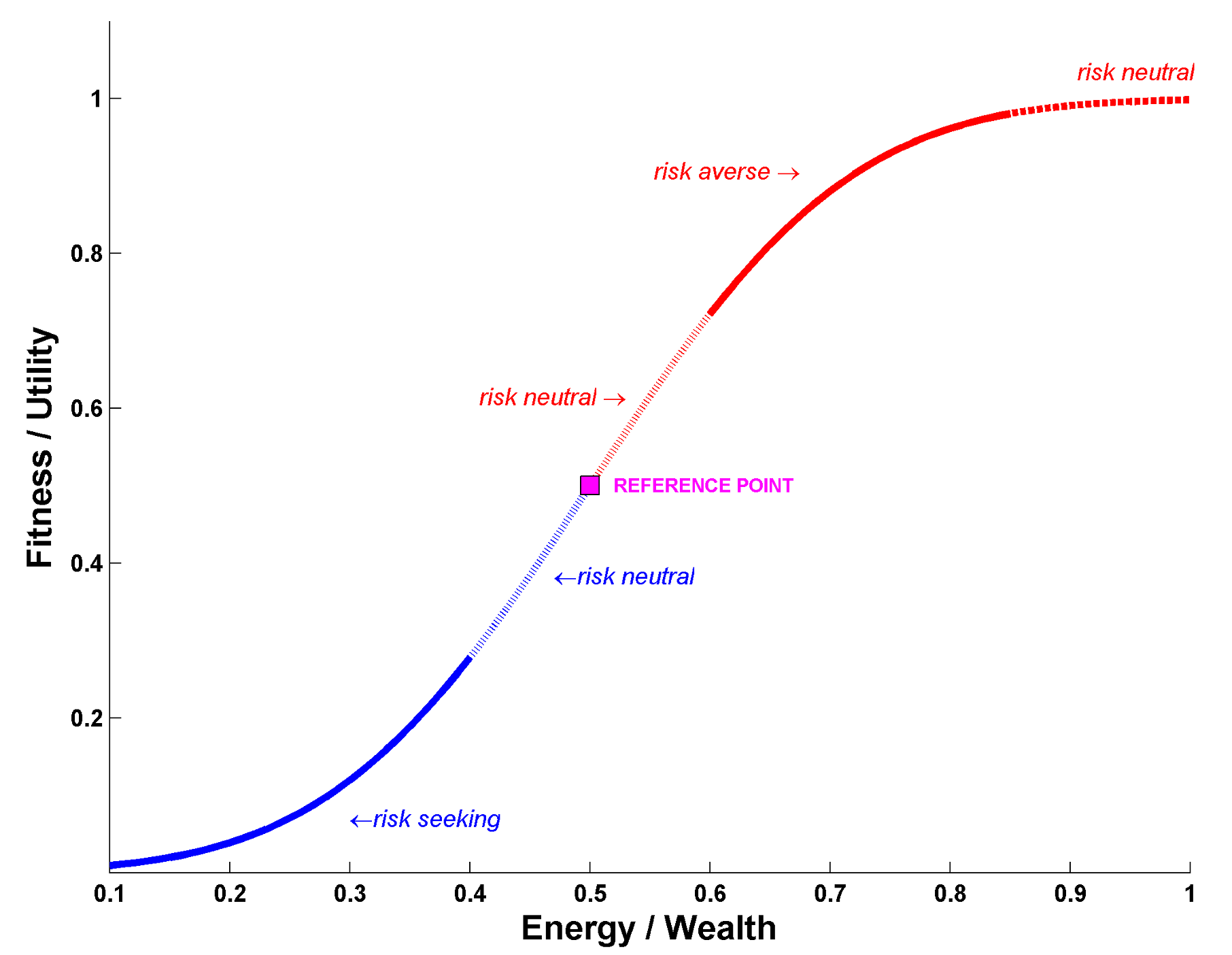

Supplement: Figure S1 — Schematic of risk-attitude changes in relation to a reference point (either for money or for food/energy). Risk-attitude equates to the curvature of this relationship. Below reference point, risk-seeking behaviour is seen. Near the reference point, decisions are risk-neutral (insensitive to risk). As energy or wealth increases, increasing risk-aversion is seen. At very high levels (e.g. repletion or satiation), this relation saturates and we again see risk-neutral behaviour. Note: Above the reference point, the relationship between wealth/energy and utility/fitness is marginally decreasing (concave), engendering risk-aversion. This is because, for a concave function, the average ‘utility’ (i.e. average y-axis value) of any two outcomes always equates to less ‘wealth’ than the average wealth (i.e. average x-axis value) of the same two outcomes, by Jensen's inequality. This means that a sure amount with equivalent value to the average (mean) wealth of two outcomes will always be preferred to a gamble with 50∶50 chance of getting one or other outcome, thus such an individual is described as being averse to risk. A similar argument applies for risk-seeking being engendered by a convex relation between wealth/energy and utility/fitness. (0.12 MB TIF) [file pone.0011090.s002.tif]
