# Supplementary material for: Metabolic State Alters Economic Decision Making under Risk in Humans
Source: PLoS One. 2010 Jun 16;5(6):e11090. doi: 10.1371/journal.pone.0011090 (PMC2886827; doi:10.1371/journal.pone.0011090)
Supplement: Table S1 — Baseline anthropometric and glucose results for included subjects. (0.04 MB DOC) [file pone.0011090.s003.doc]

**Table S1: Baseline anthropometric and glucose results for included subjects.**

| **Age (yrs)** | **BMI**  **(kg/m^2^)** | **Body fat (%)** | **Glucose (mmol/L)** |
| --- | --- | --- | --- |
| 22 | 24.5 | 14.0 | 4.9 |
| 20 | 22.2 | 12.5 | 4.9 |
| 22 | 20.7 | 10.5 | 4.5 |
| 32 | 21.2 | 11.0 | 5.2 |
| 20 | 24.7 | 16.5 | 4.9 |
| 20 | 21.6 | 12.5 | 4.5 |
| 25 | 21.2 | 11.5 | 4.8 |
| 22 | 22.8 | 13.0 | 4.7 |
| 22 | 20.3 | 8.5 | 4.8 |
| 22 | 20.4 | 9.5 | 4.7 |
| 27 | 25.0 | 16.0 | 4.7 |
| 22 | 20.3 | 10.0 | 4.9 |
| 21 | 21.9 | 15.5 | 4.6 |
| 22 | 23.1 | 15.0 | 4.9 |
| 23 | 25.0 | 14.5 | 4.5 |
| 34 | 24.8 | 19.0 | 4.5 |
| 46 | 23.3 | 11.5 | 5.1 |
| 20 | 22.9 | 12.0 | 4.8 |
