# Supplementary material for: Metabolic State Alters Economic Decision Making under Risk in Humans
Source: PLoS One. 2010 Jun 16;5(6):e11090. doi: 10.1371/journal.pone.0011090 (PMC2886827; doi:10.1371/journal.pone.0011090)
Supplement: Table S2 — List of 200 lotteries used for risk preference elicitation (all amounts in pounds; risk difference in pounds2). (0.45 MB DOC) [file pone.0011090.s004.doc]

**Table S2: List of 200 lotteries used for risk preference elicitation (all amounts in pounds; risk difference in pounds^2^).**

| ***Lottery A*** | | | | ***Lottery B*** | | | | ***Expected value difference*** | ***Risk difference*** |
| --- | --- | --- | --- | --- | --- | --- | --- | --- | --- |
| **Card 1** | **Card 2** | **Card 3** | **Card 4** | **Card 1** | **Card 2** | **Card 3** | **Card 4** |
| 20 | 20 | 20 | 20 | 0 | 0 | 20 | 100 | 10 | 2266.67 |
| 40 | 40 | 40 | 40 | 0 | 60 | 60 | 60 | 5 | 900.00 |
| 60 | 60 | 60 | 60 | 40 | 60 | 100 | 100 | 15 | 900.00 |
| 20 | 20 | 40 | 60 | 0 | 0 | 40 | 100 | 0 | 1866.67 |
| 0 | 20 | 20 | 40 | 0 | 0 | 0 | 80 | 0 | 1333.33 |
| 40 | 40 | 40 | 80 | 0 | 0 | 100 | 100 | 0 | 2933.33 |
| 20 | 40 | 60 | 80 | 0 | 20 | 40 | 100 | -10 | 1200.00 |
| 0 | 20 | 20 | 20 | 0 | 0 | 20 | 60 | 5 | 700.00 |
| 20 | 20 | 20 | 40 | 0 | 20 | 80 | 100 | 25 | 2166.67 |
| 60 | 60 | 60 | 60 | 20 | 60 | 60 | 100 | 0 | 1066.67 |
| 40 | 40 | 40 | 80 | 0 | 0 | 0 | 100 | -25 | 2100.00 |
| 60 | 60 | 60 | 60 | 0 | 20 | 20 | 100 | -25 | 1966.67 |
| 80 | 80 | 80 | 80 | 60 | 100 | 100 | 100 | 10 | 400.00 |
| 0 | 20 | 40 | 60 | 20 | 20 | 100 | 100 | 30 | 1466.67 |
| 0 | 0 | 80 | 80 | 20 | 20 | 40 | 100 | 5 | -700.00 |
| 20 | 20 | 20 | 20 | 0 | 60 | 60 | 80 | 30 | 1200.00 |
| 0 | 20 | 40 | 60 | 0 | 0 | 60 | 100 | 10 | 1733.33 |
| 0 | 0 | 0 | 60 | 0 | 40 | 40 | 100 | 30 | 800.00 |
| 20 | 40 | 40 | 100 | 0 | 40 | 60 | 100 | 0 | 533.33 |
| 20 | 40 | 80 | 80 | 0 | 0 | 0 | 100 | -30 | 1600.00 |
| 40 | 40 | 40 | 80 | 0 | 80 | 80 | 80 | 10 | 1200.00 |
| 60 | 80 | 80 | 80 | 0 | 40 | 60 | 100 | -25 | 1633.33 |
| 60 | 60 | 60 | 60 | 0 | 0 | 0 | 80 | -40 | 1600.00 |
| 40 | 40 | 40 | 40 | 0 | 40 | 60 | 60 | 0 | 800.00 |
| 20 | 20 | 20 | 20 | 0 | 0 | 60 | 100 | 20 | 2400.00 |
| 0 | 20 | 40 | 40 | 20 | 20 | 40 | 100 | 20 | 1066.67 |
| 20 | 40 | 80 | 80 | 0 | 60 | 80 | 100 | 5 | 966.67 |
| 60 | 80 | 100 | 100 | 40 | 100 | 100 | 100 | 0 | 533.33 |
| 20 | 40 | 40 | 40 | 20 | 20 | 60 | 100 | 15 | 1366.67 |
| 40 | 40 | 100 | 100 | 20 | 60 | 100 | 100 | 0 | 266.67 |
| 20 | 20 | 20 | 20 | 0 | 40 | 40 | 40 | 10 | 400.00 |
| 0 | 0 | 40 | 40 | 20 | 40 | 60 | 100 | 35 | 633.33 |
| 20 | 40 | 60 | 80 | 0 | 0 | 60 | 100 | -10 | 1733.33 |
| 20 | 60 | 60 | 100 | 0 | 0 | 80 | 100 | -15 | 1700.00 |
| 0 | 0 | 40 | 40 | 20 | 20 | 80 | 100 | 35 | 1166.67 |
| 20 | 20 | 20 | 20 | 0 | 100 | 100 | 100 | 55 | 2500.00 |
| 0 | 20 | 20 | 40 | 0 | 0 | 0 | 60 | -5 | 633.33 |
| 40 | 40 | 40 | 40 | 0 | 20 | 60 | 80 | 0 | 1333.33 |
| 20 | 20 | 20 | 40 | 0 | 0 | 60 | 80 | 10 | 1600.00 |
| 20 | 20 | 20 | 20 | 0 | 60 | 100 | 100 | 45 | 2233.33 |
| 60 | 60 | 60 | 60 | 40 | 40 | 40 | 80 | -10 | 400.00 |
| 20 | 20 | 20 | 60 | 0 | 80 | 100 | 100 | 40 | 1866.67 |
| 0 | 60 | 60 | 60 | 20 | 40 | 40 | 100 | 5 | 300.00 |
| 20 | 40 | 40 | 60 | 0 | 60 | 80 | 100 | 20 | 1600.00 |
| 20 | 20 | 20 | 60 | 0 | 0 | 40 | 60 | -5 | 500.00 |
| 0 | 0 | 20 | 20 | 0 | 0 | 0 | 60 | 5 | 766.67 |
| 80 | 80 | 80 | 80 | 40 | 40 | 100 | 100 | -10 | 1200.00 |
| 80 | 80 | 80 | 80 | 20 | 20 | 80 | 100 | -25 | 1700.00 |
| 80 | 80 | 80 | 80 | 40 | 60 | 100 | 100 | -5 | 900.00 |
| 40 | 40 | 40 | 40 | 0 | 0 | 20 | 60 | -20 | 800.00 |
| 80 | 80 | 80 | 80 | 0 | 0 | 20 | 100 | -50 | 2266.67 |
| 0 | 60 | 60 | 60 | 40 | 60 | 60 | 80 | 15 | -633.33 |
| 40 | 40 | 40 | 40 | 20 | 40 | 40 | 40 | -5 | 100.00 |
| 0 | 0 | 20 | 40 | 0 | 0 | 0 | 80 | 5 | 1233.33 |
| 60 | 80 | 80 | 80 | 0 | 100 | 100 | 100 | 0 | 2400.00 |
| 0 | 0 | 0 | 60 | 20 | 40 | 40 | 40 | 20 | -800.00 |
| 20 | 20 | 40 | 40 | 0 | 20 | 100 | 100 | 25 | 2633.33 |
| 40 | 40 | 40 | 40 | 20 | 40 | 80 | 80 | 15 | 900.00 |
| 0 | 20 | 40 | 40 | 0 | 0 | 20 | 80 | 0 | 1066.67 |
| 0 | 0 | 40 | 40 | 0 | 0 | 20 | 100 | 10 | 1733.33 |
| 20 | 20 | 40 | 60 | 0 | 40 | 40 | 80 | 5 | 700.00 |
| 0 | 40 | 40 | 40 | 20 | 40 | 60 | 80 | 20 | 266.67 |
| 60 | 60 | 60 | 60 | 40 | 40 | 80 | 100 | 5 | 900.00 |
| 0 | 0 | 60 | 60 | 0 | 20 | 40 | 100 | 10 | 666.67 |
| 0 | 20 | 20 | 60 | 0 | 0 | 60 | 80 | 10 | 1066.67 |
| 0 | 40 | 40 | 40 | 0 | 0 | 60 | 60 | 0 | 800.00 |
| 20 | 20 | 40 | 40 | 0 | 80 | 80 | 100 | 35 | 1833.33 |
| 20 | 20 | 20 | 20 | 0 | 20 | 80 | 80 | 25 | 1700.00 |
| 20 | 40 | 80 | 80 | 20 | 20 | 80 | 100 | 0 | 800.00 |
| 0 | 0 | 80 | 80 | 40 | 40 | 40 | 100 | 15 | -1233.33 |
| 0 | 40 | 40 | 40 | 20 | 60 | 60 | 100 | 30 | 666.67 |
| 60 | 60 | 60 | 60 | 40 | 40 | 80 | 80 | 0 | 533.33 |
| 0 | 20 | 40 | 40 | 20 | 20 | 80 | 100 | 30 | 1333.33 |
| 20 | 20 | 20 | 40 | 0 | 0 | 100 | 100 | 25 | 3233.33 |
| 0 | 0 | 40 | 60 | 0 | 20 | 40 | 60 | 5 | -233.33 |
| 60 | 60 | 60 | 60 | 20 | 80 | 100 | 100 | 15 | 1433.33 |
| 20 | 20 | 20 | 20 | 0 | 0 | 0 | 60 | -5 | 900.00 |
| 20 | 20 | 20 | 20 | 0 | 60 | 60 | 60 | 25 | 900.00 |
| 0 | 60 | 60 | 60 | 0 | 40 | 40 | 80 | -5 | 166.67 |
| 20 | 20 | 40 | 60 | 0 | 20 | 20 | 80 | -5 | 833.33 |
| 20 | 20 | 20 | 40 | 0 | 0 | 60 | 60 | 5 | 1100.00 |
| 0 | 0 | 0 | 60 | 40 | 40 | 100 | 100 | 55 | 300.00 |
| 0 | 0 | 40 | 40 | 20 | 60 | 80 | 80 | 40 | 266.67 |
| 0 | 20 | 20 | 60 | 0 | 0 | 80 | 100 | 20 | 2133.33 |
| 20 | 40 | 40 | 60 | 20 | 20 | 40 | 100 | 5 | 1166.67 |
| 0 | 60 | 60 | 60 | 20 | 20 | 80 | 80 | 5 | 300.00 |
| 20 | 40 | 80 | 80 | 0 | 0 | 80 | 100 | -10 | 1866.67 |
| 0 | 20 | 20 | 20 | 0 | 0 | 60 | 80 | 20 | 1600.00 |
| 20 | 40 | 40 | 40 | 0 | 40 | 40 | 60 | 0 | 533.33 |
| 0 | 20 | 20 | 40 | 0 | 0 | 80 | 100 | 25 | 2500.00 |
| 60 | 80 | 80 | 80 | 20 | 60 | 80 | 100 | -10 | 1066.67 |
| 60 | 60 | 60 | 60 | 0 | 0 | 0 | 100 | -35 | 2500.00 |
| 20 | 40 | 80 | 80 | 40 | 60 | 60 | 100 | 10 | -266.67 |
| 0 | 20 | 20 | 20 | 0 | 0 | 0 | 60 | 0 | 800.00 |
| 0 | 0 | 40 | 60 | 0 | 0 | 80 | 100 | 20 | 1866.67 |
| 20 | 20 | 20 | 40 | 0 | 0 | 0 | 60 | -10 | 800.00 |
| 0 | 0 | 0 | 60 | 0 | 0 | 20 | 80 | 10 | 533.33 |
| 60 | 60 | 80 | 80 | 0 | 80 | 100 | 100 | 0 | 2133.33 |
| 0 | 0 | 20 | 20 | 0 | 20 | 20 | 60 | 15 | 500.00 |
| 20 | 20 | 40 | 40 | 0 | 0 | 40 | 80 | 0 | 1333.33 |
| 20 | 40 | 40 | 60 | 0 | 20 | 80 | 100 | 10 | 2000.00 |
| 0 | 0 | 40 | 40 | 20 | 40 | 60 | 60 | 25 | -166.67 |
| 0 | 0 | 0 | 60 | 40 | 80 | 100 | 100 | 65 | -100.00 |
| 20 | 40 | 40 | 40 | 0 | 80 | 80 | 80 | 25 | 1500.00 |
| 20 | 20 | 20 | 40 | 0 | 0 | 20 | 80 | 0 | 1333.33 |
| 20 | 20 | 20 | 60 | 0 | 0 | 0 | 100 | -5 | 2100.00 |
| 0 | 40 | 40 | 40 | 20 | 20 | 60 | 60 | 10 | 133.33 |
| 0 | 20 | 40 | 40 | 0 | 0 | 80 | 80 | 15 | 1766.67 |
| 20 | 40 | 40 | 40 | 0 | 0 | 40 | 60 | -10 | 800.00 |
| 60 | 60 | 80 | 80 | 20 | 100 | 100 | 100 | 10 | 1466.67 |
| 0 | 20 | 40 | 60 | 20 | 20 | 40 | 60 | 5 | -300.00 |
| 0 | 40 | 40 | 40 | 20 | 40 | 40 | 100 | 20 | 800.00 |
| 60 | 60 | 60 | 60 | 40 | 60 | 80 | 80 | 5 | 366.67 |
| 20 | 20 | 20 | 60 | 0 | 40 | 40 | 60 | 5 | 233.33 |
| 0 | 0 | 0 | 60 | 20 | 20 | 80 | 100 | 40 | 800.00 |
| 20 | 40 | 60 | 80 | 0 | 40 | 60 | 100 | 0 | 1066.67 |
| 20 | 20 | 40 | 40 | 0 | 0 | 20 | 60 | -10 | 666.67 |
| 0 | 20 | 40 | 60 | 20 | 20 | 80 | 100 | 25 | 1033.33 |
| 0 | 0 | 0 | 40 | 20 | 20 | 100 | 100 | 50 | 1733.33 |
| 0 | 0 | 20 | 20 | 0 | 0 | 20 | 60 | 10 | 666.67 |
| 60 | 60 | 60 | 60 | 0 | 0 | 60 | 80 | -25 | 1700.00 |
| 40 | 40 | 40 | 80 | 0 | 80 | 80 | 100 | 15 | 1566.67 |
| 40 | 40 | 40 | 80 | 20 | 20 | 80 | 100 | 5 | 1300.00 |
| 0 | 20 | 20 | 20 | 0 | 0 | 60 | 100 | 25 | 2300.00 |
| 20 | 40 | 40 | 40 | 0 | 0 | 60 | 80 | 0 | 1600.00 |
| 0 | 60 | 60 | 60 | 20 | 20 | 80 | 100 | 10 | 800.00 |
| 60 | 60 | 60 | 60 | 0 | 40 | 60 | 80 | -15 | 1166.67 |
| 0 | 0 | 80 | 80 | 40 | 60 | 100 | 100 | 35 | -1233.33 |
| 20 | 20 | 20 | 60 | 0 | 0 | 80 | 100 | 15 | 2366.67 |
| 20 | 40 | 40 | 100 | 0 | 60 | 100 | 100 | 15 | 1033.33 |
| 20 | 40 | 40 | 60 | 0 | 100 | 100 | 100 | 35 | 2233.33 |
| 20 | 20 | 40 | 40 | 0 | 0 | 0 | 100 | -5 | 2366.67 |
| 20 | 20 | 40 | 40 | 20 | 20 | 20 | 100 | 10 | 1466.67 |
| 40 | 40 | 40 | 40 | 20 | 60 | 80 | 80 | 20 | 800.00 |
| 20 | 20 | 20 | 40 | 0 | 40 | 80 | 80 | 25 | 1366.67 |
| 60 | 60 | 80 | 80 | 20 | 40 | 40 | 100 | -20 | 1066.67 |
| 0 | 0 | 40 | 60 | 0 | 0 | 20 | 100 | 5 | 1366.67 |
| 20 | 20 | 20 | 40 | 0 | 60 | 80 | 100 | 35 | 1766.67 |
| 0 | 20 | 40 | 40 | 0 | 0 | 0 | 60 | -10 | 533.33 |
| 60 | 60 | 60 | 60 | 40 | 100 | 100 | 100 | 25 | 900.00 |
| 0 | 60 | 60 | 60 | 0 | 0 | 20 | 100 | -15 | 1366.67 |
| 0 | 0 | 80 | 80 | 40 | 100 | 100 | 100 | 45 | -1233.33 |
| 20 | 40 | 40 | 40 | 0 | 20 | 60 | 60 | 0 | 800.00 |
| 0 | 20 | 40 | 40 | 20 | 20 | 60 | 100 | 25 | 1100.00 |
| 60 | 60 | 80 | 80 | 0 | 0 | 80 | 100 | -25 | 2633.33 |
| 20 | 40 | 40 | 40 | 0 | 20 | 20 | 60 | -10 | 533.33 |
| 0 | 0 | 40 | 40 | 0 | 0 | 20 | 80 | 5 | 900.00 |
| 60 | 80 | 80 | 80 | 0 | 80 | 80 | 100 | -10 | 1866.67 |
| 0 | 0 | 80 | 80 | 40 | 80 | 100 | 100 | 40 | -1333.33 |
| 0 | 60 | 60 | 60 | 0 | 20 | 20 | 80 | -15 | 300.00 |
| 0 | 40 | 40 | 40 | 20 | 20 | 20 | 80 | 5 | 500.00 |
| 60 | 60 | 80 | 80 | 0 | 40 | 80 | 100 | -15 | 1833.33 |
| 40 | 40 | 40 | 40 | 0 | 20 | 60 | 60 | -5 | 900.00 |
| 20 | 20 | 20 | 20 | 0 | 20 | 40 | 40 | 5 | 366.67 |
| 0 | 60 | 60 | 60 | 40 | 40 | 40 | 80 | 5 | -500.00 |
| 0 | 20 | 20 | 40 | 0 | 0 | 40 | 100 | 15 | 1966.67 |
| 20 | 20 | 20 | 60 | 0 | 20 | 80 | 80 | 15 | 1300.00 |
| 0 | 20 | 20 | 20 | 0 | 0 | 20 | 40 | 0 | 266.67 |
| 20 | 20 | 40 | 60 | 0 | 0 | 60 | 100 | 5 | 2033.33 |
| 0 | 0 | 40 | 60 | 0 | 0 | 0 | 80 | -5 | 700.00 |
| 20 | 40 | 40 | 60 | 0 | 20 | 40 | 80 | -5 | 900.00 |
| 0 | 20 | 20 | 20 | 0 | 0 | 100 | 100 | 35 | 3233.33 |
| 0 | 20 | 40 | 60 | 0 | 0 | 60 | 80 | 5 | 1033.33 |
| 20 | 40 | 40 | 40 | 0 | 40 | 60 | 80 | 10 | 1066.67 |
| 80 | 80 | 80 | 80 | 40 | 40 | 80 | 100 | -15 | 900.00 |
| 20 | 20 | 20 | 20 | 0 | 80 | 80 | 80 | 40 | 1600.00 |
| 20 | 20 | 40 | 40 | 0 | 20 | 20 | 100 | 5 | 1833.33 |
| 0 | 20 | 20 | 20 | 0 | 0 | 60 | 60 | 15 | 1100.00 |
| 20 | 20 | 40 | 40 | 0 | 60 | 80 | 80 | 25 | 1300.00 |
| 0 | 0 | 40 | 40 | 0 | 0 | 0 | 60 | -5 | 366.67 |
| 20 | 20 | 20 | 40 | 0 | 0 | 0 | 100 | 0 | 2400.00 |
| 0 | 40 | 40 | 40 | 0 | 20 | 80 | 80 | 15 | 1300.00 |
| 0 | 60 | 60 | 60 | 0 | 20 | 60 | 100 | 0 | 1066.67 |
| 20 | 60 | 60 | 100 | 20 | 20 | 80 | 100 | -5 | 633.33 |
| 20 | 20 | 20 | 40 | 0 | 60 | 60 | 60 | 20 | 800.00 |
| 20 | 20 | 40 | 40 | 0 | 0 | 0 | 60 | -15 | 766.67 |
| 0 | 20 | 40 | 40 | 20 | 20 | 100 | 100 | 35 | 1766.67 |
| 80 | 80 | 80 | 80 | 0 | 40 | 60 | 100 | -30 | 1733.33 |
| 20 | 20 | 40 | 60 | 0 | 0 | 0 | 100 | -10 | 2133.33 |
| 40 | 40 | 40 | 40 | 20 | 20 | 20 | 60 | -10 | 400.00 |
| 20 | 40 | 40 | 40 | 0 | 0 | 0 | 100 | -10 | 2400.00 |
| 0 | 20 | 20 | 20 | 0 | 0 | 0 | 40 | -5 | 300.00 |
| 60 | 80 | 80 | 80 | 60 | 80 | 100 | 100 | 10 | 266.67 |
| 20 | 20 | 20 | 20 | 0 | 0 | 60 | 80 | 15 | 1700.00 |
| 40 | 40 | 40 | 40 | 0 | 40 | 40 | 80 | 0 | 1066.67 |
| 20 | 20 | 40 | 40 | 0 | 0 | 60 | 80 | 5 | 1566.67 |
| 80 | 80 | 80 | 80 | 60 | 80 | 80 | 100 | 0 | 266.67 |
| 20 | 20 | 20 | 20 | 0 | 0 | 60 | 60 | 10 | 1200.00 |
| 20 | 20 | 20 | 20 | 0 | 0 | 40 | 40 | 0 | 533.33 |
| 20 | 20 | 20 | 40 | 0 | 0 | 40 | 40 | -5 | 433.33 |
| 0 | 60 | 60 | 60 | 40 | 80 | 80 | 80 | 25 | -500.00 |
| 20 | 20 | 20 | 20 | 0 | 0 | 20 | 60 | 0 | 800.00 |
| 0 | 0 | 20 | 20 | 0 | 0 | 0 | 40 | 0 | 266.67 |
| 20 | 40 | 40 | 40 | 0 | 0 | 80 | 80 | 5 | 2033.33 |
| 0 | 60 | 60 | 60 | 0 | 0 | 0 | 80 | -25 | 700.00 |
| 20 | 20 | 40 | 60 | 0 | 40 | 100 | 100 | 25 | 2033.33 |
| 20 | 40 | 40 | 40 | 0 | 60 | 60 | 100 | 20 | 1600.00 |
| 0 | 0 | 0 | 60 | 40 | 40 | 40 | 100 | 40 | 0.00 |
| 20 | 40 | 40 | 60 | 0 | 20 | 20 | 80 | -10 | 933.33 |
| 80 | 80 | 100 | 100 | 20 | 100 | 100 | 100 | -10 | 1466.67 |
