# Supplementary material for: Metabolic State Alters Economic Decision Making under Risk in Humans
Source: PLoS One. 2010 Jun 16;5(6):e11090. doi: 10.1371/journal.pone.0011090 (PMC2886827; doi:10.1371/journal.pone.0011090)
Supplement: Appendix S1 — Decision making model. (0.02 MB DOC) [file pone.0011090.s005.doc]

**Appendix S1**

To obtain an absolute measure of risk-preference, we applied the following logistic regression model to each subject’s choices:

$P(choice)= \frac{1}{1+e^{-\beta\Delta U}}$ $\Delta U = \Delta\mu- \rho\Delta\sigma^{2}$

***where***

Δ*σ^2^* is the difference in variance of the lotteries, Δ*μ* is the difference in EV of the lotteries, and *ρ* is a free parameter (risk coefficient) that indicates the EV-risk trade-off for that subject. *β* is a free parameter indicating choice randomness, or sensitivity to the stimulus dimension (i.e. utility difference, ΔU). The risk coefficient indicates the increase in mean value difference (in pounds sterling) required to offset a one [pound sterling]^2^ increment in variance difference of an option. This model partitions choices into riskier/less risky choices according to a probabilistic decision rule that accounts for intrinsic noisy choice, according to a one-dimensional softmax function typically used in decision analysis [44], identical to a multiple logistic regression constrained to fit through the origin (i.e. we assume that if there is no utility difference, choice is indifferent between the two options). Using this formalism, we then can make separate inferences regarding the noise and risk-return parameters. We estimated model parameters with maximum-likelihood analysis, using a non-linear Nelder-Mead simplex search algorithm.
